# Predicting postoperative vomiting among orthopedic patients receiving patient-controlled epidural analgesia using SVM and LR

**DOI:** 10.1038/srep27041

**Published:** 2016-06-01

**Authors:** Hsin-Yun Wu, Cihun-Siyong Alex Gong, Shih-Pin Lin, Kuang-Yi Chang, Mei-Yung Tsou, Chien-Kun Ting

**Affiliations:** 1 Department of Anesthesiology, Shuang Ho Hospital, Taipei Medical University (No. 291, Zhongzheng Rd., Zhonghe, Taipei 23561,Taiwan.; 2 Department of Anesthesiology, School of Medicine, College of Medicine, Taipei Medical University, Taipei, Taiwan; 3 Department of Electrical Engineering, Chang Gung University, Taoyuan County 33302, Taiwan; 4 Portable Energy System Group, Green Technology Research Center, College of Engineering, Chang Gung University, Taoyuan County 33302, Taiwan; 5 Chang Gung Memorial Hospital, Taoyuan 33304, Taiwan; 6 Department of Anesthesiology, Taipei Veterans General Hospital and National Yang-Ming University, 11217, Taiwan

## Abstract

Patient-controlled epidural analgesia (PCEA) has been applied to reduce postoperative pain in orthopedic surgical patients. Unfortunately, PCEA is occasionally accompanied by nausea and vomiting. The logistic regression (LR) model is widely used to predict vomiting, and recently support vector machines (SVM), a supervised machine learning method, has been used for classification and prediction. Unlike our previous work which compared Artificial Neural Networks (ANNs) with LR, this study uses a SVM-based predictive model to identify patients with high risk of vomiting during PCEA and comparing results with those derived from the LR-based model. From January to March 2007, data from 195 patients undergoing PCEA following orthopedic surgery were applied to develop two predictive models. 75% of the data were randomly selected for training, while the remainder was used for testing to validate predictive performance. The area under curve (AUC) was measured using the Receiver Operating Characteristic curve (ROC). The area under ROC curves of LR and SVM models were 0.734 and 0.929, respectively. A computer-based predictive model can be used to identify those who are at high risk for vomiting after PCEA, allowing for patient-specific therapeutic intervention or the use of alternative analgesic methods.

Vomiting is one of the most frequent adverse effects of patient-controlled epidural analgesia (PCEA)^1^,^2^. Post-operative vomiting has found to have a strong negative impact on patient satisfaction with PCEA and willingness to undergo such treatment. Clinical studies have shown that routine use of prophylactic medication to prevent vomiting has little advantage to simply treating vomiting while it’s occurring^3^,^4^. The use of prophylactic anti-emetic medication is frequently not recommended because of a lack of patient satisfaction, potential side effects, and economic factors^4^,^5^. As a result, it is important to accurately identify patients with a high risk of vomiting during PCEA, thus helping anesthetists determine the optimum PCEA dosage and ensure adequate patient understanding. Various models and methods, such as logistic regression or Cox regression, have been applied to investigate the risk factors involved with postoperative nausea and vomiting (PONV). Applying logistic regression to the same dataset previously used for LR and ANN showed that artificial neural networks are a better method for predicting PONV for orthopedic patients receiving PCEA^6^,^7^. However, support vector machine (SVM), a supervised machine learning algorithm, can also be used to detect complex patterns within data sets. It is usually used for classification and regression and has been applied to many studies for computer-aided diagnosis, outcome prediction, and signal processing^8^,^9^,^10^. No previous studies have applied SVM to investigate PONV, particularly for PCEA cases. We suspect that SVM could prove to be a more powerful method for predicting PONV for orthopedic patients using PCEA.

We designed a retrospective study to construct an effective predictive model for PONV with high sensitivity, specificity, positive predictive value (PPV), and negative predictive value (NPV). The proposed prediction model for PCEA could potentially be used to adjust the background agent and infusion rate of PCEA in response to individual patient conditions to prevent PONV. The predictive model could be used to identify patients who are at high risk of vomiting after PCEA, allowing for early intervention to reducing potential discomfort and anxiety while increasing patient satisfaction. We also compared the predictive performance of the SVM model with that of a model constructed using logistic regression.

## Methods and Materials

### Study population

This retrospective study was conducted under the approval of the Institutional Review Board (VGHIRB No.: 96-10-07A) at TAIPEI VETERAS GENERAL HOSPITAL. Epidural PCA profiles of orthopedic cases were collected from January to March 2007. The sample group included patients with postoperative PCEA following operations involving the lower extremities. After discarding cases with incomplete PCA records or missing demographic data, we obtained 195 cases. According to the dermatomal level of incision site, epidural catheters were set prior to surgery using an 18-gauge Touhy needle and a 20-gauge epidural catheter. To identify the epidural space, a loss-of-resistance technique was used, and the epidural catheter was placed 5–7 cm into the epidural space. All epidural catheters were tested before surgery to ensure adequate function. The sample group also excluded cases of intrathecal or intravascular migration. All orthopedic surgeries were performed using spinal-epidural anesthesia with hyperbaric 0.5% bupivacaine 12 to 15 mg for spinal anesthesia and a loading dose 6 to 10 ml of 0.25% bupivacaine with 5 μg/ml fentanyl for epidural anesthesia.

The patient-controlled analgesia device (AIM PLUS SYSTEM, Abbott Laboratories, North Chicago, IL, USA) was connected to patient’s epidural catheter on arrival at the post-anesthesia care unit. An analgesic agent of bupivacaine (0.0625%) and fentanyl (1 μg/ml), was applied to all patients. Initial PCEA settings included a background infusion rate of 3–5 ml/h with a PCEA bolus of 2 ml and a lockout interval of 20–30 min. Inadequate analgesia was defined by numerical rating score (NRS) > 5 (0 = no pain and 10 = worst possible pain). An additional loading dose with 5-ml of the infusion mixture was given in response to inadequate analgesia with an increase of 1–2 ml/h of the background infusion rate. The patient controlled analgesia (PCA) charts documented the continuous infusion and the cumulative dose after machine setup. The PCA team staff visited all patients at least once a day (morning or afternoon), or whenever necessary. The PCEA dose was adjusted in response to any PCEA-related complaints, such as numbness, nausea, vomiting, pruritus, or other adverse effects. Based on the severity of complaints, the PCEA continuous dose was decreased by 1–2 ml; all complaints and changes were recorded.

The final sample included 195 participants for model construction and performance evaluation:

1. Patient demographic attributes: Age, gender, height, weight, body mass index (BMI).
2. Operation-related and PCEA-related attributes: Type of surgery, bolus epidural dose of PCA, epidural catheter insertion level, epidural catheter length in the epidural space, vomiting.

#### Logistic Regression (LR)

SPSS (SPSS for Windows, version V15, SPSS Inc.) was used for data and statistical analysis. The data set used is the same as in a previous article about LR and artificial neural networks^6^. The model was constructed using a random selection of 75% (n = 146) of the total data set, while the remaining 25% (n = 49) were used as a test set to validate the predictive performance. Mean value, standard deviation, and 95% confidence interval were calculated as metric variables. We used nonparametric independent t-test to analyze metric variables between the training and testing sets. Categorical variables were assessed for a significant association by either Chi-Square statistics or student *t*-test. Variable selection was also applied using the Forward selection algorithm. It automatically selects variables for inclusion of exclusion by calculating their respective contributions to the model. At each step, each variable that is not in the model is tested for inclusion in the model. The most significance of these variables is added so long as its P-value is below certain pre-set level. Therefore, the algorithm begins by including the variable that is most significant in the initial analysis, followed by continuously adding variables until none of the remaining variables are “significant” when added to the model. Analysis was terminated when no further variables were available for inclusion. Moreover, logistic regression was applied to estimate the coefficients (β) of these variables. In summary, applying the logistic equation to these results allowed us to estimate the probability of vomiting.

The modeling of the vomiting probability by logistic regression can be written as the following equation:

Assume a binary random variable Y as the vomiting status (1 indicating vomiting; 0 no vomiting). The probability for vomiting is noted as


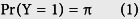


and the probability for no vomiting is


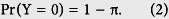


Nine possible explanatory variables (e.g., gender, age, total knee replacement, etc.) are noted as *X*_1_, *X*_2_, *X*_3_, … *X*_9_. The logistic regression assumes the effect of the explanatory variables as a linear combination, so that the model is written as


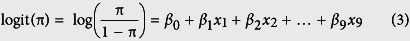


*β*_0_, *β*_1_, …, *β*_9_ are the regression coefficients of the regression model. The logistic regression estimated the regression coefficients from the data and the effects of the explanatory variables on vomiting probability were expressed as an odds ratio. Taking the explanatory variable *X*_1_ for example, the corresponding effect on vomiting is 
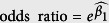
. The crude odds ratio is only used as a single explanatory variable in the model, while the adjusted odds ratio is used to add more than one variable in the model to allow for the effect to be adjusted by another explanatory variable.

#### Support Vector Machine (SVM)

The Support Vector Machine (SVM) is a statistically supervised learning approach method, used for classification and regression. It classifies data by finding a best hyper-plane which can separate the point data of one class from another with the largest margin. One SVM classifier can be built by a kernal function and some parameter. We developed the SVM model using the NeuroSolutions for Excel (Version 5.0, NEURODIMENSION Inc.). Due to limitations imposed by the NEURODIMENSION, we were unable to tune the relaxation parameter or the kernel function in the SVM model.

The formulation of SVM is shown as follows:

There are N training data points {(x_1_, y_1_), (x_2_, y_2_), (x_3_, y_3_) … (x_N_, y_N_)}, where *x*_*i*_ ∈  *R*^d^ and *y*_*i*_ ∈ {+1, −1}. We want to find a linear separating hyper-plane classifier as the equation.


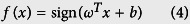


In addition, we want a hyper-plane with a maximum separating margin between the two classes.

Distance between 2 points:


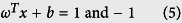



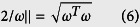



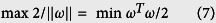


Data which are not linearly separable may allow training errors.


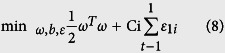



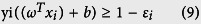



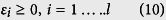


If ε > 1, x_i_ is not at the correct site of the separating plane. Therefore we use the cross-validation to find the best C-value to construct the model. The training epochs were set to 2000 and the step size was 0.01. As mentioned in the LR section, 75% data were applied to the training process, and the remaining 25% were used as a test set to validate the predictive performance. Following completion of the training process, the SVM were tested with the same remaining patients (n = 49) who were not selected for training and whose outcome regarding occlusion was unknown to the SVM.

#### Performance Evaluation

The predictive performance was evaluated using the test data. Furthermore, we tried to valid the models by calculating the sensitivity, specificity, accuracy (i.e., the number of correct predictions divided by total predictions), positive predictive value (PPV), negative predictive value (NPV), and likelihood ratios for positive and negative predictions. The receiver operating characteristic (ROC) curve was measured for the discriminating power of the prediction models. ROC analysis provides tools for optimal model selection, with suboptimal models discarded independently from the cost context or the class distribution. The area under the ROC curve (AUC) was applied to measure discriminatory power. In our study, we selected the cut-off value corresponding to the highest accuracy (minimal false negative and false positive results) for the sensitivity and specificity of each prediction tool.

## Results

The analyses are based on retrospectively collected data of 195 patients (91 men and 104 women) who were classified into vomiting and non-vomiting groups according to their response to PCEA. Similar to previous studies^6^,^7^, the data was classified as continuous data and categorical data (Table 1), which are respectively expressed as mean with standard deviation (SD) and the count with percentage. We used the Independent t-test and Chi-square test to compare patient characteristics and variables related to PCEA usage of the two groups. Table 2 shows the demographic data and characteristics related to PCEA usage. There is no significant difference between the training and testing groups. The incidence of vomiting is about 30.6% for orthopedic patients receiving PCEA (49.0% for female and 7.7% for male).

**Figure 1:**
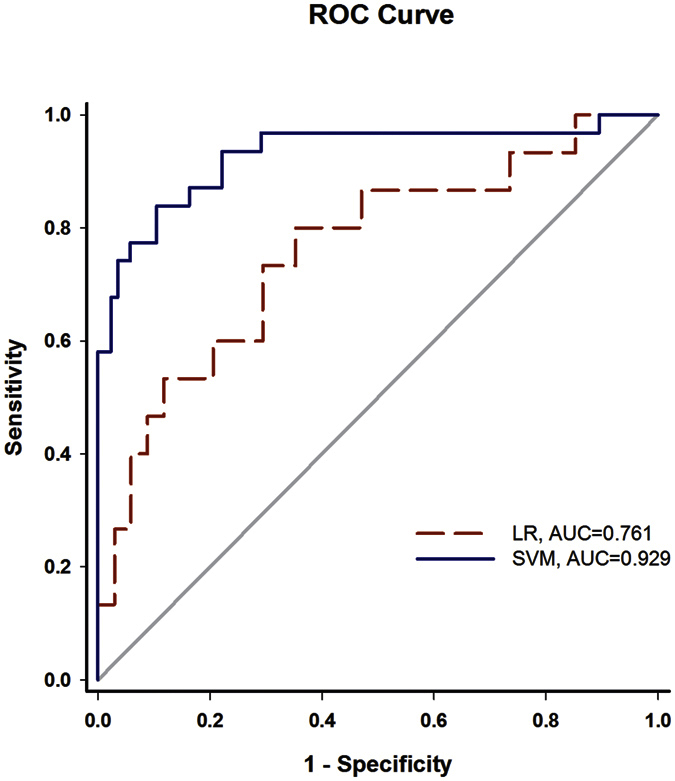
ROC curves of logistic regression and SVM.

The final logistic regression model includes three variables: sex, catheter length in epidural space, and type of operation. The probability of vomiting can also be derived by the regression coefficient of the LR model. The crude and adjusted odds ratio (OR) of the potential risk factors related to vomiting induced by PCEA are presented in Table 3. Female gender, catheter length in epidural space, and type of orthopedic surgery are associated with vomiting during PCEA. The most significant factor related to vomiting is gender (crude OR = 15.356, 95% CI: 5.098–46.256). Compared to gender, catheter length and type of orthopedic surgery were protective factors in PCEA-related vomiting. Other factors did not have a statistically significant effect on vomiting. Female sex is still the most significant risk factor related to PCEA-induced vomiting after forward model selection. The adjusted OR and its 95% CI of female patients are 55.880 and 11.505~271.420, respectively. Other risk factors after adjustment include catheter length in epidural space (OR = 0.294, 95% CI: 0.151~0.570) and type of orthopedic surgery (OR = 0.321, 95% CI: 0.101~1.022). Figure 1 shows the receiver operative characteristic (ROC) curves. The results indicate that the SVM models have better discriminating power than the LR model to identify patients with a high risk for vomiting during PCEA after orthopedic surgery.

## Discussion

Our findings indicated that, similar to previous studies, the incidence of vomiting was about 30.6% for orthopedic patients receiving PCEA (49.0% for female and 7.7% for male)^1^,^11^,^12^,^13^, where the analyses are different from our previous works^6^,^7^. Many factors may affect the incidence of vomiting. For instance, fentanyl-based PCEA may result in a lower incidence of vomiting than morphine-based treatment^14^. Given the widespread use of PCEA in postoperative orthopedic patient care, establishing a good predictive tool to avoid side effects, especially vomiting, extremely is extremely important^1^,^6^,^15^. Such a model would allow for the early identification of high risk patients, allowing for the early implementation of prevention strategies, thereby reducing the incidence of vomiting.

We investigated the SVM and LR models to detect whether or not vomiting occurred after PCEA, and summarized the findings of the multivariate analysis using ROC curves. An area under curve (AUC) of 73.4% has been demonstrated for the logistic regression model, where the sensitivity and specificity are 53.3% and 82.4%, respectively. By comparison, the SVM model has an AUC of 92.9% with the sensitivity of 83.9% and the specificity of 89.5%. Evidently, the SVM outperforms the other models in terms of AUC, accuracy, sensitivity, PPV, and NPV.

Recently, computer-based medical decision support systems have been applied to clinical use for medical diagnosis, decisions, and patient care^1^,^16^. The SVM model used in this study can be easily applied to any standard desktopcomputer^17^. It appears to be a suitable model for clinicians to apply reasonable and cost-effective antiemetic treatments in practice. Support vector machine is a class of learning system based on statistical learning theories. The model represents examples as points in space, mapped so that the examples of the separate categories are divided by the maximum possible gap. New examples are then mapped into the same space and their category membership is predicted based on which side of the gap they fall on. This approach can effectively recognize certain patterns once trained using a set of representative data.

A computer-based predictive model can be used to facilitate the early identification of patients at a high risk of vomiting after PCEA. Moreover, the use of such a model enables us to design patient-specific therapeutic interventions or recommend the use of alternative methods of analgesia^18^,^19^. For example, high-risk patients on PCEA can be prescribed prophylactic anti-emetics based on the predictive model.

With regard to the LR model, we identified several potential factors associated with vomiting by fitting a logistic regression using a stepwise forward selection procedure (P < 0.05 to enter). The stepwise logistic model selection found that vomiting correlated with, female gender, catheter length in epidural space and type of orthopedic surgery. The finding of the female gender risk factor supports previous findings^12^,^19^,^20^,^21^. Preventive strategies may be considered for female patients due to the strong correlation of vomiting induced by PCEA. There is an unexpected protective factor related to extended epidural catheter length. Previous studies reported that longer epidural catheter length increased the risk of intravenous insertion, intrathecal migration, knotting or unilateral sensory analgesia^22^,^23^. However, no previous studies have examined the incidence of vomiting and its correlation with epidural catheter length. We cannot explain the exact mechanism for this notable finding, and the relation between vomiting during PCEA and epidural catheter length requires further evaluation and study. Of the types of orthopedic surgery studied, total knee replacement was found to be a risk factor for vomiting with PCEA. The possible cause of this factor also is also unclear.

### Concluding Remarks

The present study is subject to certain limitations. First of all, the data is taken from a relative small number of cases, and the predictive power of the model may be better supported by the inclusion of more cases. Second, the analysis should include additional variables by using a larger sample, which may result in the predicting model being more AUC. Third, data classification is not sufficiently detailed. Our predictive model seeks to create a simple method that can be applied easily in clinical settings. Thus, we grouped all orthopedic operations with PCEA. Therefore, the resulting model may miss some procedure-specific information.

In summary, we have demonstrated the power of the SVM and LR models to predict the likelihood of vomiting with PCEA, and the models could be applied using the parameters available before PCEA implementation. The present study has certain clinical implications. This individualized model can be applied to explain the risk of vomiting in patients receiving PCEA, and patients found to be at high risk can potentially be given alternative analgesic techniques or antiemetic therapeutic intervention.

### Overview

Postoperative patients receiving PCEA frequently suffer from PONV, but it is also unacceptable in general clinical practice to continuously supply patients with antiemetics. Therefore, a means of identifying patients who are at high risk for PCEA-induced vomiting in advance would be of clear value.

No previous studies have sought to use SVM to predict the risk of PONV in orthopedic patients receiving PCEA. Our study compared this approach to results obtained using LR, based on the same database. Our results show that SVM provides significantly better accuracy in identifying the PONV group, justifying the hypothesis. The proposed model could be used clinically to effectively reduce patient discomfort and anxiety.

The data sample for this work included 195 patients who received lower limb orthopedic surgery in the first three months of 2007. We used a needle capable of loss of resistance to perform epidural anesthesia at a location determined by type of surgery. Anesthesia was maintained post-operative and in both constant dose and self-controllable dose. The results and the ROC curves shown in Fig. 1 suggest the SVM model is more reliable than the LR model for identifying PONV patients. Despite the model’s efficacy, several influential factors should be further clarified. For example, Fentanyl-based PCEA may result in less vomiting than Morphine-based PCEA. In addition, we found that gender (female) is the most important risk factor, suggesting that antiemetics should be supplied prior to the induction of PCEA for women. Moreover, the degree to which the catheter is inserted into the epidural space is evidently related to pain relief. To the best of our knowledge this is a new finding, and will be further studied in future work.

Future studies should also consider the reason why knee replacement surgery is more strongly correlated with PONV. Our study suffers from two limitations: the small sample size and lack of sufficient data regarding orthopedic surgery type. Despite these limitations, we have demonstrated the effectiveness of the LR and SVM models in predicting PONV in PCEA patients. The proposed approaches could be used to effectively identify patients at high risk for PONV prior to surgery, allowing for the implementation of antiemetic countermeasures in advance.

## Additional Information

**How to cite this article**: Wu, H.-Y. *et al*. Predicting postoperative vomiting among orthopedic patients receiving patient-controlled epidural analgesia using SVM and LR. *Sci. Rep*. **6**, 27041; doi: 10.1038/srep27041 (2016).

## Figures and Tables

**Table 1:** Variables used for training of the LR and SVM models^6^,^7^.

Variable	Age	Gender	Height	Weight	BMI
Coding	Years	1: female; 0: male	cm	kg	Body mass index
Variable	Length (catheter length in the epidural space)	Bolus dose	Total knee replacement (TKR)	Epidural level (Insertion site of EA catheter)	
Coding	cm	Ml	0: not TKR*; 1: TKR	0: above L4; 1: below L4	

^*^Surgery on other lower extremities.

**Table 2:**
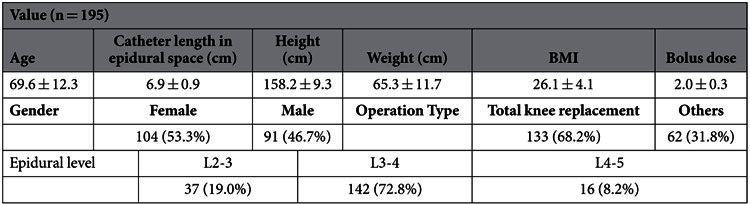
Patient characteristics^6^,^7^.

Parametric data were showed as mean with SD.

Categorical data were showed as count & percentage.

*p < 0.05.

**Table 3:** Unadjusted and adjusted OR of potential risk factors related to PCEA-induced vomiting^6^,^7^.

	Unadjusted	95% CI			Adjusted	95% CI		P-value
	OR	Lower	Upper	P value	OR	Lower	Upper	
Gender (female)	6.896	2.513	18.923	<0.001	16.345	4.555	56.647	<0.001
Age (year)	1.003	0.977	1.031	0.801				
Total knee replacement (TKR)	1.511	0.873	2.614	0.140				
Epidural level	0.315	0.091	1.093	0.069				
Length (cm)	0.506	0.325	0.785	0.002	0.467	0.305	0.716	0.001
Height (cm)	0.953	0.913	0.995	0.028	1.066	1.004	1.132	0.038
Weight (kg)	0.972	0.945	1.001	0.057				
Bolus dose (ml)	0.748	0.168	3.325	0.702				
BMI (kg/m^2^)	0.971	0.899	1.049	0.457				
