# Corrigendum: Predicting postoperative vomiting among orthopedic patients receiving patient-controlled epidural analgesia using SVM and LR

**DOI:** 10.1038/srep28408

**Published:** 2016-06-27

**Authors:** Hsin-Yun Wu, Cihun-Siyong Alex Gong, Shih-Pin Lin, Kuang-Yi Chang, Mei-Yung Tsou, Chien-Kun Ting

Scientific Reports 6: Article number: 27041; 10.1038/srep27041 published online: 06012016; updated: 06272016.

In this Article, Shih-Pin Lin is incorrectly affiliated with ‘Portable Energy System Group, Green Technology Research Center, College of Engineering, Chang Gung University, Taoyuan County 33302, Taiwan’. The correct affiliation is listed below:

Department of Anesthesiology, Taipei Veterans General Hospital and National Yang-Ming University, 11217, Taiwan.

